# A systematic review of the etiopathogenesis of Kienböck's disease and a critical appraisal of its recognition as an occupational disease related to hand-arm vibration

**DOI:** 10.1186/1471-2474-13-225

**Published:** 2012-11-21

**Authors:** Stéphane Stahl, Adelana Santos Stahl, Christoph Meisner, Afshin Rahmanian-Schwarz, Hans-Eberhard Schaller, Oliver Lotter

**Affiliations:** 1 Department of Plastic, Hand and Reconstructive Surgery, Burn Center, BG-Trauma Center, Eberhard-Karl University of Tübingen, Schnarrenbergstr. 95, Tübingen, 72076, Germany; 2 Department for Plastic Surgery, Marienhospital Stuttgart, Böheimstr. 37, Stuttgart 70199, Germany; 3 Department of Medical Biometry, Eberhard-Karl University of Tübingen, Westbahnhofstr. 55, Tübingen, 72070, Germany

**Keywords:** Lunate necrosis, Kienböck’s disease, Etiopathogenesis, Occupational disease, Systematic review

## Abstract

**Background:**

We systematically reviewed etiological factors of Kienböck’s disease (osteonecrosis of the lunate) discussed in the literature in order to examine the justification for including Kienböck’s disease (KD) in the European Listing of Occupational Diseases.

**Methods:**

We searched the Ovid/Medline and the Cochrane Library for articles discussing the etiology of osteonecrosis of the lunate published since the first description of KD in 1910 and up until July 2012 in English, French or German. Literature was classified by the level of evidence presented, the etiopathological hypothesis discussed, and the author's conclusion about the role of the etiopathological hypothesis. The causal relationship between KD and hand-arm vibration was elucidated by the Bradford Hill criteria.

**Results:**

A total of 220 references was found. Of the included 152 articles, 140 (92%) reached the evidence level IV (case series). The four most frequently discussed factors were negative ulnar variance (n=72; 47%), primary arterial ischemia of the lunate (n=63; 41%), trauma (n=63; 41%) and hand-arm vibration (n=53; 35%). The quality of the cohort studies on hand-arm vibration did not permit a meta-analysis to evaluate the strength of an association to KD. Evidence for the lack of consistency, plausibility and coherence of the 4 most frequently discussed etiopathologies was found. No evidence was found to support any of the nine Bradford Hill criteria for a causal relationship between KD and hand-arm vibration.

**Conclusions:**

A systematic review of 220 articles on the etiopathology of KD and the application of the Bradford Hill criteria does not provide sufficient scientific evidence to confirm or refute a causal relationship between KD and hand-arm vibration. This currently suggests that, KD does not comply with the criteria of the International Labour Organization determining occupational diseases. However, research with a higher level of evidence is required to further determine if hand-arm vibration is a risk factor for KD.

## Background

Kienböck’s disease (KD) is an osteonecrosis involving the lunate bone that finally results in carpal collapse and severe wrist arthrosis. Currently no evidence exists demonstrating a treatment that has the ability to lead to disease regression or even halt disease progression [1]. The numerous synonyms for KD (lunate malacia, aseptic, idiopathic, avascular or traumatic lunate necrosis) infer that the true etiology remains poorly understood. The uncertain etiology goes along with ambiguous diagnostic criteria which in turn account for the unknown incidence and prevalence. However, KD is considered a rare disease [2] (prevalence less than 5 in 10,000 people [3]).

Traditionally KD is recognized as an occupational disease caused by hand-arm vibration or by trauma in work-related injuries [4]. Low frequency hand-arm vibrations (8-50Hz) have been suggested to cause repetitive microtrauma, thereby inducing osteonecrosis of the lunate [5]. Hand-arm vibrations are commonly associated with the use of percussive tools (chipping hammer, jack hammer, large and small sand rammer, rock drill). Occupations at risk include building and maintenance of roads and railways, construction, forestry, foundries, heavy engineering, mining and quarrying. The requirement of a minimum of 2 years of exposure to vibrating tools in the performance of regular and heavy work was introduced in 1965 in Germany based on expert opinion [6]. The estimation of the vibration acceleration rate of jackhammers in the 1930s (vibration acceleration rate a_hv_=13,5m/s^2^) resulted in the implementation of further occupational preconditions for vibration-induced KD in Germany in 1998 [7]. The necessary total vibration exposure dose was estimated on the basis of the mean exposure time relayed in 59 medico-legal assessments (expert reports) of suspected occupational disease (240 working days per year; daily exposure of 5h/d; minimal duration of exposition of 2 years; total vibration exposure dose 5.122m/s^2^) [7].

KD is listed under the number 505.01 in the European Listing of Occupational Diseases in countries such as Germany and France, whereas this is not the case in others such as Austria [8]. Despite efforts toward a European Union-wide harmonization (Recommendation 2003/670/EC), reliable information regarding the recognition of KD as an occupational disease in Europe can only be obtained by contacting each individual national authority. According to the International Labour Organization (ILO), an occupational disease is “any disease contracted as a result of an exposure to risk factors arising from work activity [9]”. This definition implies causality between the disease and the exposure factor and must be confirmed with sufficient probability. The factors substantiating a causal association must outweigh those factors substantiating alternative theories.

The analytical framework of Bradford Hill's criteria (strength of association, consistency, specificity, temporality, biological gradient, biological plausibility, biological coherence, experimental evidence and analogy), represent an important tool for scientifically determining causality between any discussed factors and KD [10]. Yet the evaluation of consistency, plausibility and coherence as described by Bradford Hill requires the evaluation of all existing theories and knowledge. The trauma and the primary arterial ischemia hypothesis were first described by Kienböck in 1910, who postulated that trauma led to compromised vascularization of the lunate [11]. Since multiple reasons may account for arterial ischemia, a distinction is made between trauma and arterial ischemia. Müller was the first to presume that negative ulnar variance might cause KD secondary to an unbalanced overload of the lunate in 1920 [12]. Upon a follow-up examination of 10 patients performing heterogeneous manual labour in Germany, Müller also suspected a correlation between professional activity and disease occurrence and brought forth the first arguments in favour of recognizing KD as caused by occupational repetitive microtrauma [12]. In 1931, a higher prevalence of KD was suspected among underground workers in mines and quarries and the incorporation of KD into the German list of occupational diseases was recommended, without knowledge of the strength of association between KD and hand-arm vibrations [4].

The average percentage of recognized occupational diseases in relation to those suspected is 80% in France and Switzerland, between 40 and 50% in Sweden, Portugal, Austria and Belgium, and under 25% in Germany, Finland and Italy [13]. This low percentage may be due to (I) the lack of their clear definition and of convincing evidence for a causal relationship [13], (II) imprecise diagnostic criteria [14], and (III) the unemployment and/or fatalities associated with the resulting invalidity as is characteristic for patients with KD. We therefore conducted a systematic review using several electronic databases supplemented by manual searches of published reference lists, review articles and conference abstracts to elucidate the causal relationship between KD and the most frequently discussed hypotheses in order to examine the justification for including Kienböck’s disease in the European Listing of Occupational Diseases.

## Methods

A systematic review was conducted using the Ovid/Medline and the Cochrane database for the keywords “Kienböck's disease” and “etiology” including different spellings and synonyms (Additional file 1: Appendix) following PRISMA guidelines [15]. Since most hypotheses have been published in the pre-Medline era, the search was supplemented with additional references of indexed articles, bibliographies from university libraries, and from an extensive internet literature search as well as presentations from the International Meeting for Kienböck's Disease in Vienna (14.-15.05.10).

**Figure 1:**
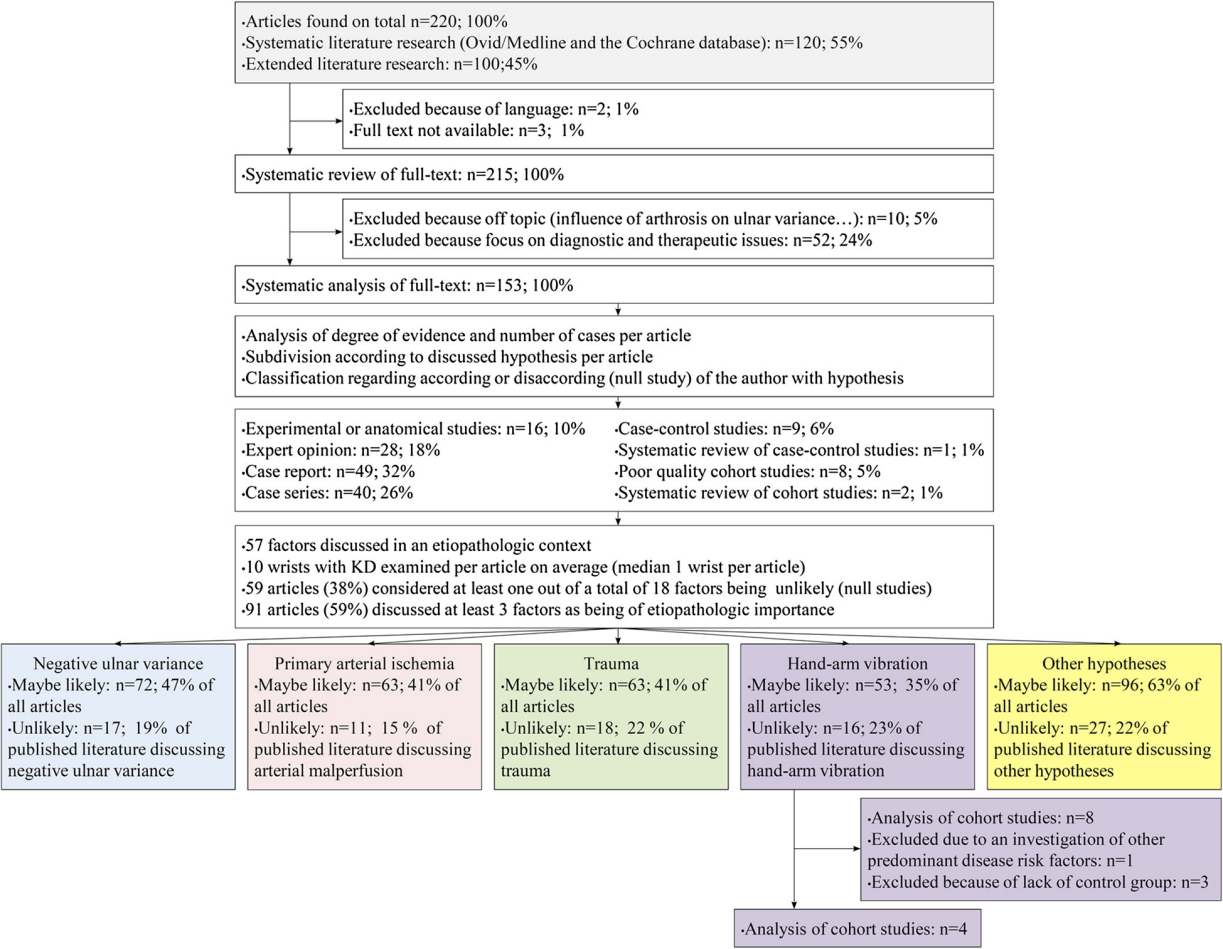
**Flow diagram demonstrating the individual steps in the study**-**selection process of literature on the causal factors of KD. **The full text of 215 articles was studied and the literature was subdivided according to the discussed etiopathological factors.

All articles discussing the etiology of KD, including predisposing and causative factors, dating from Kienböck’s initial work in 1910 [11] up until July 2012 were included in the analysis. Since every article did not include abstracts and since the etiology of KD is often discussed in different sections of a scientific article, only full-text articles were included. The PubMed research brought forth 120 articles and the extended research 100 additional articles. Two review authors independently assessed the eligibility of retrieved papers and resolved disagreements by discussion. Reasons for exclusion have been documented (Additional file 1: Appendix). All articles not published in English, French or German were excluded (n=2). The full text of three articles was not available. Articles dealing with other issues (influence of arthrosis on ulnar variance, spontaneous course of KD, osteochondritis dissecans, complication of silicone implant for KD, carpal malalignments, osteonecrosis of the scaphoid, KD classification) (n=10) were excluded, as were articles dealing exclusively with diagnostic and therapeutic aspects (n=52) (Figure 1).

Among the total of 215 full-text articles, 153 articles were included in the systematic review and screened for the discussed etiopathological factors of KD (Additional file 1: Appendix). Two authors independently reviewed all included full-text articles to identify 1) the level of evidence presented; 2) anatomic predisposing factors, occupational or mechanical risk factors and etiopathological hypotheses of KD; and 3) the author's judgment if the discussed factors and hypotheses were either likely or unlikely linked to the etiology of KD. Disagreements were resolved through consensus or by consultation with a third reviewer. The level of evidence of every article was evaluated according to the criteria of the Oxford Centre for Evidence-Based Medicine (http://www.cebm.net). Non-systematic reviews of the scientific literature were classified as expert opinions. Predisposing, risk and causative factors were categorized according to the author's judgment if they were either likely or unlikely linked to the etiology of KD while factors interpreted as mere coincidences (e.g. confounding factors) and previously unreported in the literature as being causative were not documented. Studies which found no significant results regarding the association of any of the discussed factors, or articles arguing against an etiologic role of certain factors, were defined as null studies. Since technical terms have changed over the large period of time encompassed by the study and since no clear distinction has been made along the reviewed literature, repeated microtrauma, repetitive loading, repetitive strain, cumulative trauma and hand-arm vibration were considered synonyms.

A causal relationship between hand-arm vibration and KD was evaluated according to the criteria of Bradford Hill (strength of association, consistency, specificity, temporality, biological gradient, biological plausibility, biological coherence, experimental evidence and analogy), which are widely accepted in epidemiology for investigating and defining causality and have been adopted by the International Labour Organization [9,10,16]. The evidence from the systematic literature review served as basis for the application of the Bradford Hill criteria.

## Results

### Systematic review

**Figure 2:**
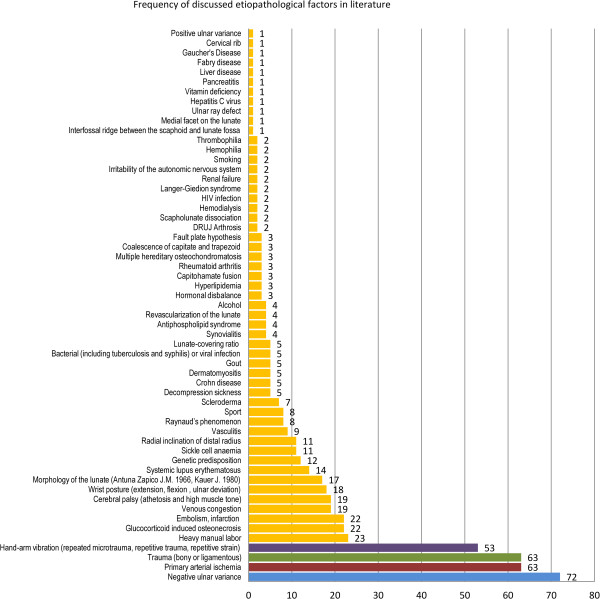
**Illustration of the frequency of discussed etiopathological factors in literature in 153 reviewed articles. **The four most frequently discussed hypotheses are marked in blue (negative ulnar variance), red (primary arterial ischemia), green (trauma) and purple (hand-arm vibration exposure); all other hypotheses are marked in yellow.

The four most frequently discussed hypotheses, negative ulnar variance (n=72 articles; 47%), primary arterial ischemia of the lunate (n=63 articles; 41%), trauma (n=63 articles; 41%) and hand-arm vibration (n=53 articles; 35%), were discussed in 124 of the 153 included articles (Figure 2). Among all reviewed articles, 91 articles (59%) supported or acknowledged at least 3 hypotheses (on average 3.72 hypotheses per article; median 3 hypotheses per article). The evidence of 140 (92%) of the relevant 152 articles reached level IV. We found 16 experimental or anatomical studies (level V), 28 expert opinions (level V), 49 case reports (≤ 3 cases; level V) and 40 case series (> 3 consecutive cases; level IV), 9 case-control (level IIIb), one systematic review of heterogeneous case-control studies (level IIIb, 8 cohort studies (level IIb) and two systematic review of heterogeneous cohort studies (level IIb). The reviewed studies on the etiopathogenesis of KD (case reports, case series, case-control and cohort studies) gathered 1528 cases of KD (median: 1 case per article, average: 10 cases per article). Altogether, we identified 57 different factors coinciding with KD that were of possible pathogenic relevance. Of the 153 full text articles included in the systematic review, 59 articles (39%) refuted at least one of these 57 hypotheses. Among the 15 most frequently discussed hypotheses that were referred to in at least 10 articles, at least 20% of published literature refuted a causal relationship for the following factors: trauma (bony or ligamentous); hand-arm vibration (repeated microtrauma, repetitive trauma, repetitive strain); embolism (infarction); genetic predisposition.

**Figure 3:**
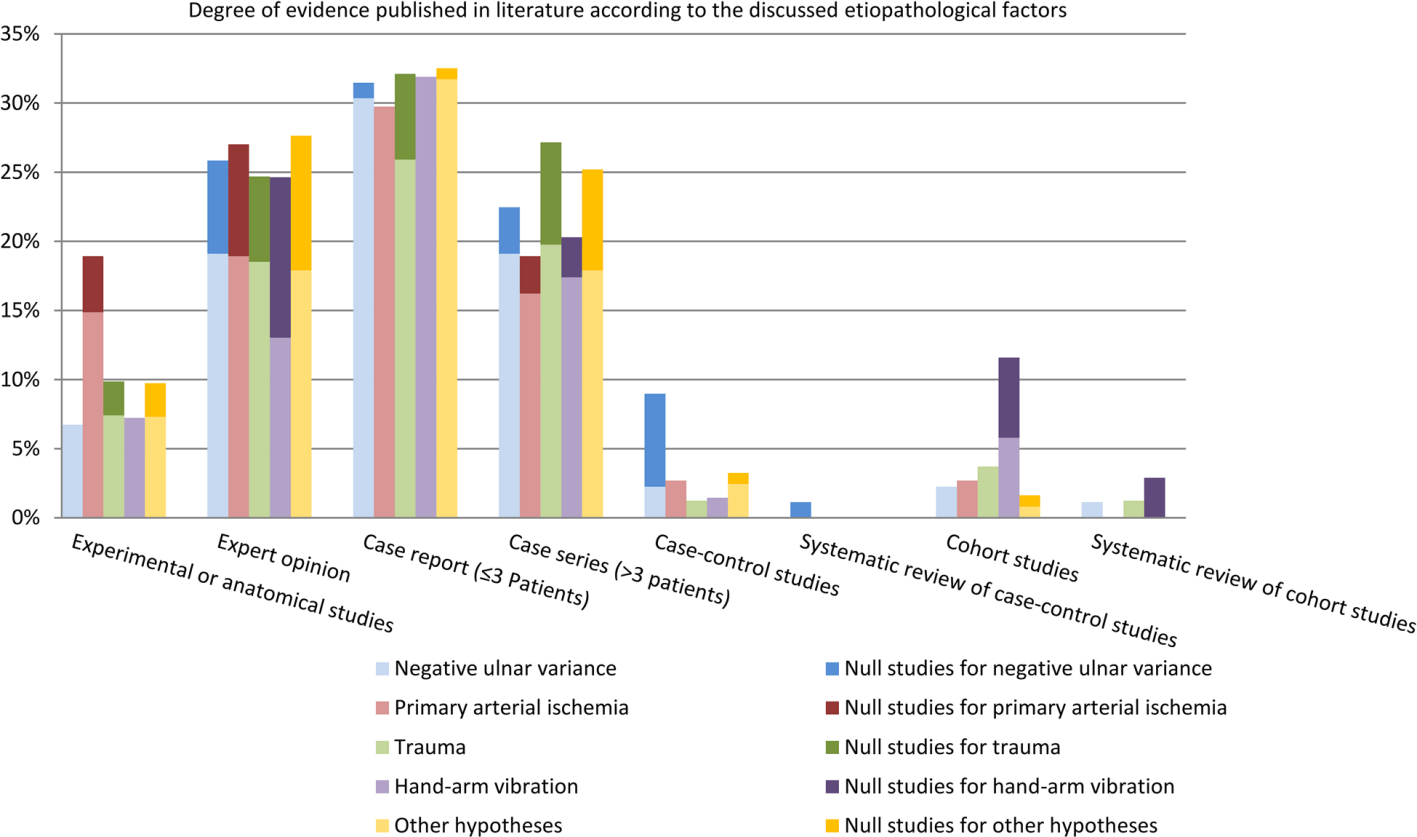
Percent distribution of the level of evidence of articles acknowledging and refuting the four most frequently discussed and other hypotheses on the etiopathology of KD.

Only 35% of the reviewed articles (53 out of a total of 153 articles) supported or suggested a causal relationship between KD and exposure to hand-arm vibration. Among these were five anatomical studies, 9 expert opinions, 22 case reports, 12 case series, one case control study and 4 cohort studies. Further, 16 studies were identified that argued against an etiopathologic role of hand-arm vibration (Figure 3). Four cohort studies in favor and four cohort studies against a causal relationship were screened to perform a meta-analysis on the strength of association between KD and hand-arm vibration. One cohort study was excluded from the review due to an investigation of other predominant disease risk factors (Additional file 1: Appendix). Three cohort studies lacked a control group (Table 1). None of the cohort studies specified the total vibration exposure dose. All of the studies used only one or two X-rays as diagnostic criteria. The largest cohort study included 580 exposed versus 90 unexposed workers. None of the previously described quality criteria to decrease susceptibility to bias as described by Sanderson et al. [17] have been met in the identified retrospective cohort studies (appropriate definition of inclusion or exclusion criteria for cohorts and controls to control selection bias; appropriate measurement methods of vibration exposure and appropriate diagnosis of KD to control incorporation bias and imperfect-standard bias; appropriate methods outlined to deal with any design-specific issues such as recall bias, interviewer bias and biased loss to follow or blinding; appropriate design and analytical methods to control confounding bias; appropriate use of statistics for primary analysis of effect to control confounding; declarations of conflict of interest or identification of funding sources).

**Table 1:** **Identification and summary of retrospective cohort studies on the incidence of KD in workers exposed and unexposed to hand**-**arm vibrations**

**Reference**	**Professional sector**	**Country**	**Number of workers**	**Number of KD cases among exposed**	**Control group**	**Diagnostic criteria**	**Anamnesis**	**Clinical examination**	**Exposition period**	**Confounding variables**	**Author**'**s conclusion regarding a causal relationship**
Decoulx P, 1957	underground workers	France	1 330	9	none	pa x-ray	none	yes	10, 13 and 13 years	5 with trauma anamnesis, 1 without exposition to vibrations	no
Roche,1961	underground workers	France	250	2	none	not specified	partially interviewed	partially	not specified	not specified	no
Kumlin, 1973	chain saw worker	Finland	35	1	age-matched from the radiological archives	pa and lat. x-ray	not specified	not specified	not specified	not specified	yes
Horváth, 1973	chain saw worker	Hungary	450	6	450 age-matched	pa x-ray	none	none	5, 7, 8 and 9 years	2 out of 6 with trauma anamnesis	yes
Laitinen, 1974	chain saw worker	Finland	359	7	none	pa x-ray	questionnaire	none	not specified	not specified	yes
Suzuki, 1978	chain saw worker	Japan	580	0	90 forestry workers	pa and lat. x-ray	none	none	not specified	not specified	no
Härkönen, 1984	chain saw worker	Finland	279	3	178 peat bog workers	pa x-ray	Interview	yes	on average 10.4 years	4 out of 5 with trauma anamnesis	no
Total:			1953	28							

Four retrospective cohort studies (purple fill color) among chain-saw workers were included in a meta-analysis with 1344 exposed and 753 unexposed workers.

Four retrospective cohort studies revealed an average incidence of KD of 0.7% (10/1344) among chain-saw workers and no KD in any of the control groups (0/753) (Fisher's Exact Test p=0.017) (Table 1).

### Bradford Hill Evaluation of Causality

#### Strength of association

The herein identified cohort studies do not permit a meta-analysis of the association of hand-arm vibration and KD since they encompass heterogeneous vibration exposures, use imprecise diagnostic criteria, do not include confounding effects or blinded radiologic evaluation. No study came up with a large enough number of cases to account for the rarity of the disease.

Taking into account the fact that KD is a rare disease, we would expect its prevalence to be much lower than 1/1,000. Assuming a prevalence of 5% among vibration exposed workers, 516 cases and 516 controls would be necessary to verify an odds ratio of 2 e.g. in a case control study design (significance level: 5%; statistical power: 80%) [18]. The number of required cases and controls would be even higher if confounding effects were also to be taken into account [19] while the calculation of the relative risk in cohort studies would require a population of several thousand. Therefore, this clearly presents the difficulties in conducting powerful cohort studies involving KD.

#### Consistency

The finding that only 35% of published literature on the etiopathology of KD favours a causal relationship between KD and hand-arm vibration, underlines the lack of scientific consensus. On the other hand, null studies regarding the association between KD and hand-arm vibration represent 10% of published literature and 23% of all articles discussing an etiopathologic role of hand-arm vibration. Four out of 7 cohort studies on the influence of hand-arm vibration conclude that there is no association. Among the 4 controlled cohort studies 2 conclude that there is no association (Table 1). In addition, the likeliness of an etiopathologic role of hand-arm vibration is less frequently discussed in literature, compared to negative ulnar variance, primary arterial ischemia or trauma (Figure 2).

#### Specificity

**Figure 4:**
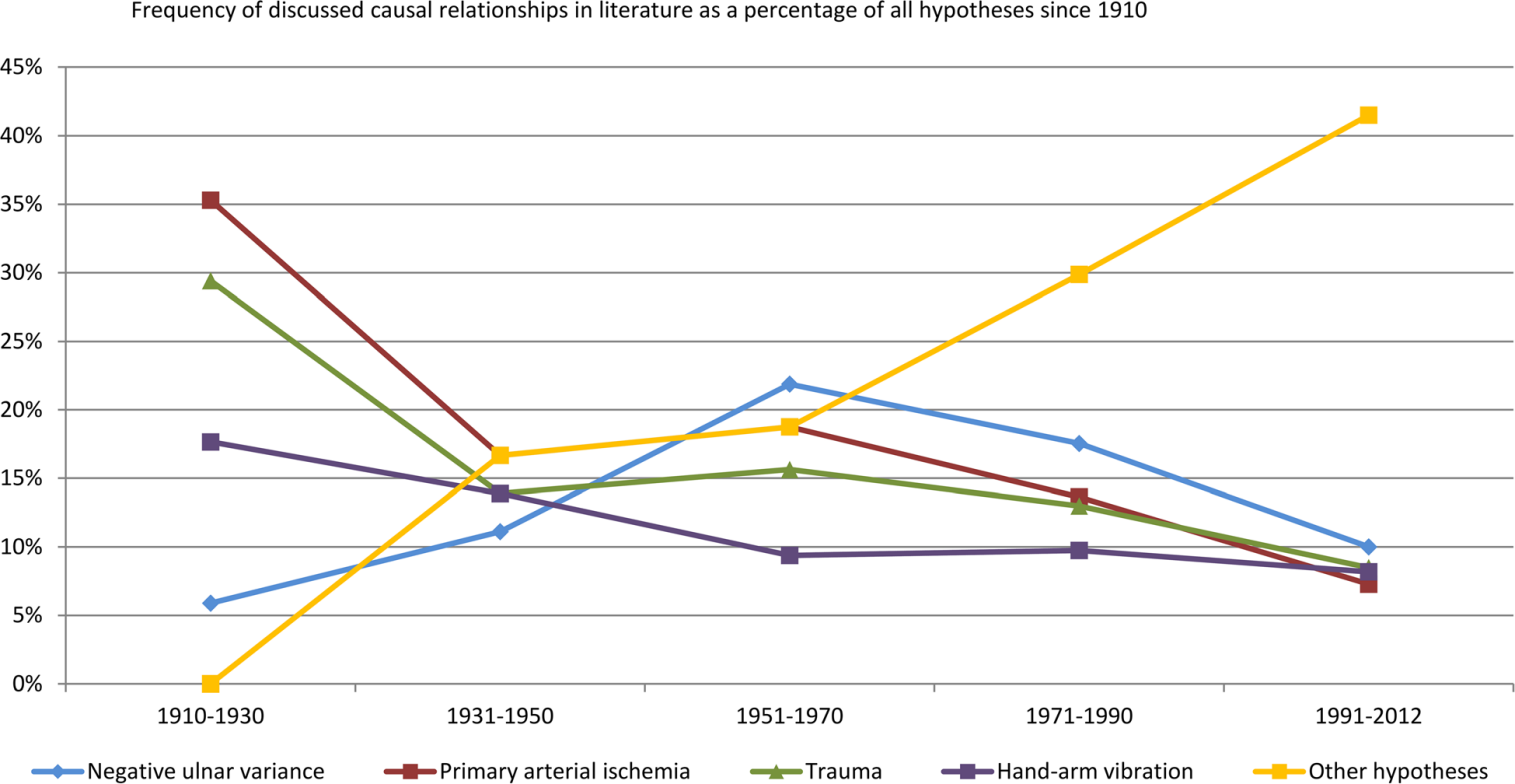
Percentages of the discussed etiopathological hypotheses in literature in relation to all the hypotheses discussed every 20 years from 1910 until July 2012.

Since 1910 the traditionally discussed explanations of the etiology of KD (negative ulnar variance, primary arterial ischemia, trauma (bony or ligamentous), hand-arm vibration (repeated microtrauma, repetitive trauma, repetitive strain)) have increasingly been replaced by other hypotheses. With regard to the popularity of the discussed etiopathological hypotheses of KD, surprisingly it was found that hand-arm vibration has not been discussed in much frequency since 1950 (Figure 4).

The hypothesis of a multifactorial genesis of KD has often been brought forward since 1936 [20-23], apparently contradicting the Bradford Hill criteria for specificity. Multivariate models and well defined cohort studies adjusting for the effects of confounding factors are required to verify this hypothesis. However to date there is no evidence indicating that KD occurs more frequently in cases of exposure to hand-arm vibrations than in populations exposed to other discussed risk factors such as negative ulnar variance, trauma, and glucocorticoid induced osteonecrosis.

#### Temporality

To establish a causal relationship, the effect must occur after the cause [10]. Occasionally patients remain clinically silent and only become symptomatic after an inciting traumatic event. Yet none of these experimental or clinical studies investigated whether the necrosis preceded the ischemia, trauma or hand-arm vibration exposure or vice versa. Negative UV is the only hypothesis that meets the criteria of temporality. Nevertheless, since negative UV is a stable condition after epiphyseal closure, the possible reasons for a delay between exposure to the risk factor and the disease occurrence must be elucidated. Methodical problems in research of rare diseases should not lead to negligence. Although proper designed prospective controlled cohort studies for rare disease demand a multi-institutional collaborative efforts and substantial funding, expert opinions, case reports and case series should not be accepted as sufficient evidence for causality.

#### Biological gradient

Since a significant association between KD and hand-arm vibration is a prerequisite to determine a biological gradient, a biological gradient regarding the effect the vibration magnitude, frequency, direction, type of tools, duration and pattern of exposure or any other extrinsic or intrinsic conditions has not been validly documented for KD.

#### Biological plausibility

Contrary to expert or to historical opinion, that “the lunate bone is the hand’s only cushion against impacts on the wrist” [24], biomechanical studies show that in a neutral position of the wrist 1/3 of the pressure is transmitted from the lunate onto the triangular fibrocartilage complex and 2/3 of the pressure onto the lunate fossa. In the working position of the wrist in ulna deviation, the lunate is however only in contact with the lunate fossa [25], and pressure is uniformly transmitted through the radiocarpal joint. Knowledge of the force transmission in the wrist would suggest osteonecrosis of the scaphoid in case of exposure to hand-arm vibration, since force transmission predominantly occurs through the scaphoid [26]. There has been no plausible explanation on why the lunate may be the only bone subject to necrosis of the 30 bones of the upper extremity in hand-arm vibration.

Exposure to low frequency vibrations has been claimed to induce inflammatory mediators that “lead to the liberation of cytolytic enzymes, disturbing the balance between cartilage removing and cartilage forming processes and thereby accelerating the degeneration of cartilage” [27]. To our knowledge no evidence has so far been found to support this hypothesis.

The German occupational disease ordinance further refers to an anatomical study from 1944 in which a mercury solution was injected into the brachial artery in neutral position with the wrist extended, observing that the lunate remained void of mercury during extension of the wrist [28]. The author's conclusion that the position of the wrist during jack-hammer work predisposes individuals to KD is not plausible since KD would be a wide-spread disease if, as suggested, an extension of the wrist would predispose to KD.

According to official epidemiologic data 1.2 million Germans are exposed to a daily vibration level greater than A(8)=2,5m/s^2^ (exposure action value in a 8 hours/day exposure), which does present a potential health risk [29]. On the basis of 4 new cases of KD recognized as an occupational disease in 2006 in Germany, the incidence can be estimated at 3:1,000,000 in exposed workers [30]. In the same year 418 new cases of KD were treated on an in-patient basis within the country's overall population of 80 million [31]. Since every patient with KD does not necessarily receive in-patient treatment, the incidence must exceed 5:1,000,000. These approximate figures infer that the incidence of KD is higher in populations without exposure to hand-arm vibrations rather than with exposure. Several explanations are possible: (I) patients with KD were treated twice per year on an in-patient basis, (II) under-reporting, (III) a healthy worker effect [32], (IV) hand-arm vibrations are not a risk factor for KD.

The vast majority of authors describe a rich and constant palmar and dorsal vascularization of the lunate bone [33] which even in cases of complete de-vascularization does not undergo necrosis [34]. Therefore, malperfusion seems to be rather the consequence than the cause of KD. Of the many known risk factors for infarction (Raynaud’s phenomenon, antiphospholipid syndrome, sickle cell anemia, decompression sickness, smoking, hypertension, atherosclerosis) none has been shown to be significantly associated with KD. Moreover, there is no evidence that anticoagulants used in thrombotic disorders may be of value for KD.

Regarding the hypothesis of trauma, Wette notes “that uncomplicated reductions of lunate dislocation never display signs of osteonecrosis, not even in cases where the lunate had not been reduced (…) severe and direct wrist strain, leading to intra-articular distal radius fracture and to fractures of the perilunar carpal bones (…) never caused late secondary lesions of the lunate bone (…) Since we have never seen such cases of theoretically possible occurrences among our patients, we must as experts defend the point of view that the fracture theory is a hypothesis for which direct evidence is lacking” [4]. Case reports of KD after perilunate and fracture dislocations are rare and their categorization is based on the observation of a hypersclerosis of the lunate in conventional X-rays [35] or a signal alteration, as it is characteristic for post-traumatic wrist MRI. Transient hypersclerosis of the lunate is well known in perilunate dislocations and should not be confused with KD [35-38]. Even spontaneous palmar dislocation of the lunate in rheumatoid arthritis does not necessarily lead to osteonecrosis [39].

#### Biological coherence

The ability of bone to respond to mechanical stimuli has been known for over a century. Moreover we now know that (I) bone preferentially responds to dynamic rather than static stimuli, (II) only short durations of loading are necessary to initiate an adaptive response, and (III) bone cells accommodate to customary mechanical loading environments [40]. Daily exposure to high-frequency whole body vibration over 1 year has shown to increase femoral trabecular bone density by 32% in adult ewes with closer spacing of bone trabeculae, which is consistent with stronger bone [41]. Progressive mechanical loading results in adaptive bone strengthening [42], where as an abrupt increase in the duration or intensity of mechanical loading may result in fatigue fracture [43]. To our knowledge no experimental evidence exists to date to conclude that a defined vibration magnitude, frequency, direction or exposure time may induce osteonecrosis.

#### Experimental evidence

According to Hill, the decreasing incidence of lung cancer in a population that stops smoking adds to the evidence of a causal relationship. By today's occupational safety and health guidelines (exposure limit value a_hv(8)_ 5m/s^2^, 220 exposure days per year) the risk of exceeding the vibration magnitude is 5 times less compared to the exposure to jackhammers in the 1930s [44]. Nevertheless an increase in the recognition of KD as an occupational disease can be seen between 2002 and 2006, despite the legal enforcement of preventive measures [45], although according to Hill the elimination of the exposure or agent should decrease disease incidence and while no evidence has been found to suggest that awareness or diagnostic criteria of KD have contributed to the increase.

#### Analogy

No evidence has been found in favor of a common cause of KD and osteonecrosis of the proximal fragment of scaphoid fractures or stress fractures.

## Discussion

The etiopathology is of paramount importance for the treatment, prognosis and, if work-related, for the prevention of KD as for many other diseases [46,47]. The purpose of this paper was to investigate the causal relationship between KD and hand-arm vibrations. This study is the first to systematically review literature on the various etiologies of KD previously presented and to evaluate evidence from 215 articles regarding the hypothesized causes of KD in light of the Bradford Hill criteria (strength of association, consistency, specificity, temporality, biological gradient, biological plausibility, biological coherence, experimental evidence and analogy) [10].

The International Labour Organization specifies that the criteria for identifying and recognizing occupational diseases need to be based on a critical review of all the available evidence, which should include strength of association, consistency, specificity, temporality or time sequence, biological gradient, biological plausibility, coherence and intervention studies [9]. No valid association of hand-arm vibration and KD was found among the reviewed literature to sustain that hand-arm vibration represents a predisposing or risk factor for KD. Using the Bradford Hill evaluation of causality, the current investigation does not support hand-arm vibration as causative of KD.

A tendency was noticeable to echo or agree with pre-existing hypotheses unless the evidence presented was above average. This tendency to agree bias might explain why the four most common hypotheses on the etiology of KD are over-represented in the literature. The arguable assumption of equivalency between different terms in the reviewed literature like hand-arm vibration, repeated microtrauma, repetitive loading, cumulative trauma and repetitive strain might have lead to an overestimation of its acknowledgement in the literature. Yet the absence of a more precise ergonomic definition of chronic strain, trauma or vibration exposure in the reviewed literature is likely due to the lack of evidence regarding its etiopathologic role in KD.

There was almost perfect agreement between two reviewers regarding the determination of 1) the level of evidence presented (98%; 3 disagreements in 153 articles); 2) the predisposing factors, risk factors and etiopathological hypotheses of KD discussed per article (95%; 7 disagreements in 153 articles); and 3) the author's judgment if the discussed factors and hypotheses were maybe or unlikely linked to the etiology of KD (99%; 2 disagreements in 153 articles). The high inter-rater agreement supports the reliability of the approach. On the other hand the attribution of explicit but also broad criteria (1: author supporting or acknowledging the discussed hypotheses, 2: author refuting the presented hypothesis) might have led to an overestimation of consistency because of a tendency of authors to echo pre-existing hypotheses.”

Our study had several limitations, most of which are inherent to any systematic review and meta-analysis of literature. Publication bias may distort meta-analysis because editors, reviewers or researchers may not want to challenge prevailing paradigms [48-51]. The preconception of what the result should look like may influence the data obtained through research [52-54]. However, this was prevented by establishing detailed protocol and inclusionary and exclusionary criteria prior to the initiation of the study. Also, it has been found that research with negative or null results is more than twice as likely to remain unpublished than studies with statistically significant results [55], and is published relatively slow compared to research with positive results [56]. The studies that found no significant results regarding the association between any of the discussed factors and KD, which also include the authors who estimated the discussed factors etiopathologically irrelevant, account for only 10% of the reviewed literature. However, studies regarding an association between negative ulnar variance and KD had a significantly higher level of evidence for “positive” studies (Wilcoxon test, p=0.038) (Figure 3). Additional research of the published evidence of the etiopathological hypotheses originally formulated between 1910 and 1926 were necessary to compensate for the limitations of the Pubmed database. Seventy percent of relevant literature from 1990 until 2012 was found in Pubmed, yet 65% of the relevant literature from 1910 until 1989 was found after an extended literature search. This is due to incomplete or absent electronic databases before 1990 and to a more precise manual search not based on keyword search but on studies abstracts and full-text articles. Used in combination, these methods help to ensure that all relevant literature is accounted for, therefore minimizing retrieval bias [57].

## Conclusion

Arbitrary, technical requirements and the exposure limit value as well as expert and historical opinions and hypothesis make it difficult to determine a causal relationship between KD and hand-arm vibration.

Limited evidence was found to sustain that hand-arm vibration represent a predisposing risk or causative factor for KD. Independent of the significance, correlations do not suffice to determine causality. Despite certain limitations, several of Bradford Hill’s criteria when taken together do contribute to a more comprehensive causal theory [10]. The summarized application of these criteria after the systematic review and meta-analysis of 220 articles demonstrates inconclusive results identifying a direct cause of KD and that to date limited evidence supports the hypothesis that KD is caused by hand-arm vibration.

The negative impact of speculative causal associations (association between power lines and cancer [58], silicone breast implants and rheumatologic illness [59], mobile phones and brain tumors [60]), urges us to carefully reflect data using the appropriate methodological safeguards and statistical tools. The examination of the Bradford Hill criteria regarding the relationship of occupational risk factors and carpal tunnel syndrome [61], occupational physical activity and low back pain [62] may challenge our preconceptions by finding conflicting evidence to support causal relationship. Moreover it has been reported that Workers’ Compensation Board claim adversely impacts outcome among individuals with low back pain [63] and carpal tunnel syndrome [47].

Meanwhile, the practice in expert reports has not changed despite or because of the many contradictions. When a trauma of certain intensity has been clearly defined and the temporal connection has been ascertained, then the causal relationship will be recognized, whilst with some reservation and the stipulation that at the moment other evidence based causes cannot be taken into account. Yet the principle of “benefit of the doubt” does not apply to scientific expert reports or to the European Listing of Occupational Diseases [64].

## Competing interests

The authors declare that they have no conflict of interest. No funding or grants from any commercial source have been received in support of the research or preparation of the work for this study.

## Authors’ contribution

Each author has contributed significantly to, and is willing to take public responsibility for, one or more aspects of the study: its design, data acquisition, and analysis and interpretation of data. All authors have been actively involved in the drafting and critical revision of the manuscript, and each provided final approval of the version to be published.

## Source of funding

There was no direct funding of this study by any commercial source.

## Pre-publication history

The pre-publication history for this paper can be accessed here:

http://www.biomedcentral.com/1471-2474/13/225/prepub

## Supplementary Material

Additional file 1 **Appendix. **List of analyzed literature with reference, level of evidence and discussed hypothesis (1: author supporting or acknowledging the discussed hypotheses, 2: author refuting the presented hypothesis). The numbers at the bottom represent the sum of articles supporting or acknowledging the discussed hypotheses. Click here for file
