# Correction: A systematic review of the etiopathogenesis of Kienböck’s disease and a critical appraisal of its recognition as an occupational disease related to hand-arm vibration

**DOI:** 10.1186/s12891-023-06850-8

**Published:** 2024-03-13

**Authors:** Stéphane Stahl, Adelana Santos Stahl, Christoph Meisner, Afshin Rahmanian-Schwarz, Hans-Eberhard Schaller, Oliver Lotter

**Affiliations:** 1 Department of Plastic, Hand and Reconstructive Surgery, Burn Center, BGTrauma Center, Eberhard-Karl University of Tübingen, Schnarrenbergstr. 95, 72076 Tübingen, Germany; 2 Department for Plastic Surgery, Marienhospital Stuttgart, Böheimstr. 37, 70199 Stuttgart, Germany; 3 Department of Medical Biometry, Eberhard-Karl University of Tübingen, Westbahnhofstr. 55, 72070 Tübingen, Germany

**Correction:** ***BMC Musculoskelet Disord*** **13, 225 (2012)**

10.1186/1471-2474-13-225

Following the publication of the original article [1], the wrong Additional file 1: Appendix (Supplementary material 1) was originally published with this article; it has now been replaced with the correct file (Supplementary material 2).

The original article [1] has been updated.

### Electronic Supplementary Material

Below is the link to the electronic supplementary material.

Supplementary material 1: The incorrect Additional file 1

Supplementary material 2: The correct Additional file 1: Appendix
